# Attribute Pair-Based Visual Recognition and Memory

**DOI:** 10.1371/journal.pone.0009571

**Published:** 2010-03-05

**Authors:** Masahiko Morita, Shigemitsu Morokami, Hiromi Morita

**Affiliations:** 1 Department of Intelligent Interaction Technology, Graduate School of Systems and Information Engineering, University of Tsukuba, Tsukuba, Ibaraki, Japan; 2 Faculty of Letters, Aichi Shukutoku University, Nagakute, Aichi, Japan; 3 Graduate School of Library, Information and Media Studies, University of Tsukuba, Tsukuba, Ibaraki, Japan; University of Sydney, Australia

## Abstract

**Background:**

In the human visual system, different attributes of an object, such as shape, color, and motion, are processed separately in different areas of the brain. This raises a fundamental question of how are these attributes integrated to produce a unified perception and a specific response. This “binding problem” is computationally difficult because all attributes are assumed to be bound together to form a single object representation. However, there is no firm evidence to confirm that such representations exist for general objects.

**Methodology/Principal Findings:**

Here we propose a paired-attribute model in which cognitive processes are based on multiple representations of paired attributes. In line with the model's prediction, we found that multiattribute stimuli can produce an illusory perception of a multiattribute object arising from erroneous integration of attribute pairs, implying that object recognition is based on parallel perception of paired attributes. Moreover, in a change-detection task, a feature change in a single attribute frequently caused an illusory perception of change in another attribute, suggesting that multiple pairs of attributes are stored in memory.

**Conclusions/Significance:**

The paired-attribute model can account for some novel illusions and controversial findings on binocular rivalry and short-term memory. Our results suggest that many cognitive processes are performed at the level of paired attributes rather than integrated objects, which greatly facilitates the binding problem and provides simpler solutions for it.

## Introduction

When we see an object, e.g., a falling red apple, its shape, color, and direction of motion are processed separately by different populations of neurons. This leads to the so-called binding problem [1], [2], i.e., how such separate attributes are integrated by us to produce a unified perception, eliciting a specific action. This question remains an important, unsolved issue in cognitive neuroscience. According to the standard theory of feature integration [3], by focusing attention on the object, all attributes of an object are integrated into a unified representation for higher cognitive processing. Such object representations containing all attributes or “object files” [4] are explicitly or implicitly assumed in most cognitive models, and efforts have been directed toward elucidating the binding mechanisms underlying them. However, most potential mechanisms involve some serious computational difficulties such as combinatorial explosion, and there seems no possible mechanism that can resolve all the difficulties. In this case, the presuppositions of the problem would require reconsideration.

Although psychological and physiological evidence [5]–[11] strongly support the existence of feature binding, they do not directly support the existence of unified representations of all attributes. For example, visual short-term memory stores bound features rather than individual features, but studies conflict as to whether an integrated object is the unit of memory [11]–[14]. From a computational viewpoint, integrating all attributes into a single representation is generally far more difficult than integrating two attributes. This not only applies to the cardinal or “grandmother” cell representation, but also to binding by the synchronous firing of neurons [1], [15] if we consider synchrony detection [16]. It should also be noted that in our daily life, conjunctions of two attributes are often essential to our cognition or action selection; however, presumably we rarely experience a problem such that conjunctions of three or more attributes are essential to solve it; that is, most problems seem solvable by focusing on a single pair or a few pairs of attributes.

Accordingly, we hypothesized that a unified representation of all attributes is not formed for an arbitrary object with more than two attributes and developed a paired-attribute model in which cognitive processes are based on multiple representations of paired attributes and their interactions. According to this model, a falling red apple is demonstrated as three separate representations: a red apple, a falling apple, and the color red falling. Conversely, predominance of these representations leads to the recognition of the falling red apple.

Our hypothesis does not deny that more than two attributes are integrated and recognized as a unified object, but it distinguishes such integration from binding of feature pairs: The former is indirect, is subsequent to the latter, and does not involve a unified representation that can compete or cooperate with other representations and can directly evoke an arbitrary response; whereas, the latter is rapid, in some cases occurring in rather early stages [9], and involves a unified representation that can operate as the basic unit of interactions. In this paper, we do not refer to the former as “binding.” We also do not deal with “intra-attribute binding,” or feature integration within a single attribute.

Although currently no evidence has been reported against our hypothesis, it is not supported by any direct evidence either. Here we explore the validity of our hypothesis by testing some predictions generated by the paired-attribute model.

## Results

### Experiment 1

A simple prediction of the paired-attribute model is that simultaneous activation of attribute-pair representations will produce a perception of a unified object. However, it is difficult to objectively validate this prediction under ordinary conditions. Thus, we performed Experiment 1 using binocular rivalry, which was also intended to verify another prediction that rivalry between incompatible attribute-pair representations is a major cause of visual competition.

**Figure 1:**
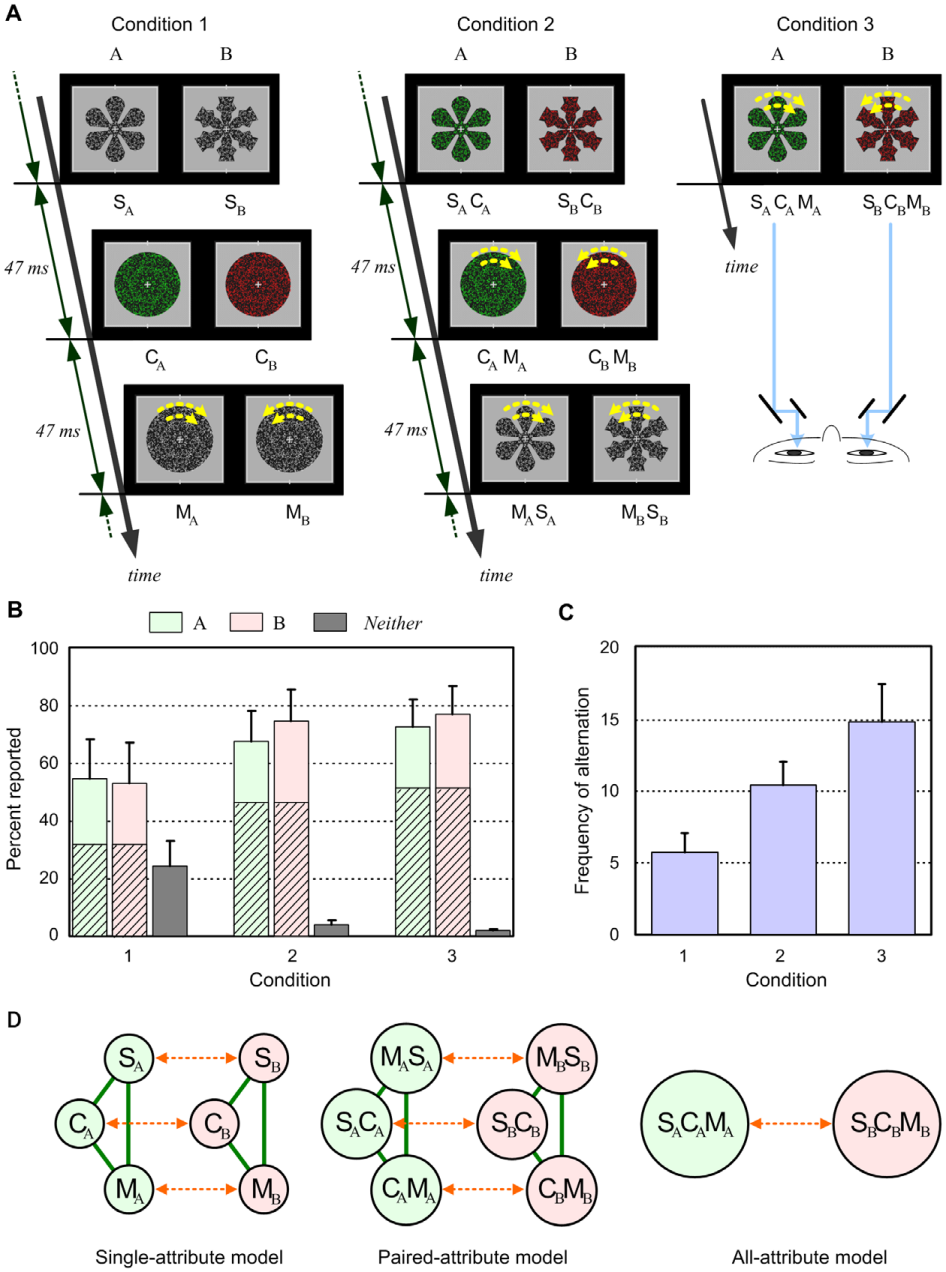
Experiment and models on rivalry between multiattribute objects. (A) Stimuli used in Experiment 1. Ten subjects with normal vision reported their perceptions by pressing buttons. (B) Mean percentage of the total period in which button A, B, or neither was pressed. Hatched bars indicate that both stimuli were perceived in different fields (mosaic dominance). Error bars indicate s.e.m. (*n* = 10). (C) Mean frequency of perceptual alternation between two stimuli during a single trial (60 s). Error bars indicate s.e.m. (D) Three hypothetical models for representations of multiattribute objects.

In this experiment, different stimuli were presented to each eye of normal human subjects (Figure 1A). Stimuli A and B contained features of three-attribute objects: A (clockwise-rotating green flower shape) and B (counterclockwise-rotating red snow shape). Strong binocular rivalry [17] occurred when all attributes were continuously presented (condition 3) and object A or B was alternately perceived.

If a series of two-attribute displays was repeatedly presented (condition 2), observers reported the same view as in condition 3, except that the background was perceived to flicker. Binocular rivalry also occurred in a similar manner (Figure 1B). Although the mean frequency of alternation decreased (Figure 1C), the difference from condition 3 was not significant (*P*>0.05). In contrast, if displays containing single attributes were presented (condition 1), observers reported that indistinct objects were perceived. The total period during which neither stimulus was perceived increased for all subjects, and alternation frequency was significantly lower than those in conditions 2 (*P* = 0.012) and 3 (*P* = 0.004), implying weaker binocular rivalry.

These results are consistent with the paired-attribute model, in which each object is represented by three units facilitating one another, and competition occurs in respective attribute pairs (Figure 1D). Two- or three-attribute stimuli (conditions 2 and 3) can sufficiently activate the units, but single-attribute stimuli (condition 1) cannot. However, the results do not necessarily exclude the single-attribute and all-attribute models in which competition occurs at the individual attribute level and the integrated whole-object level, respectively.

### Experiment 2

An exclusive prediction of the paired-attribute model is that an illusory object with three or more attributes can be perceived through erroneous integration of paired attributes. We explored this possibility using model simulations and obtained a concrete prediction that rapid serial presentation of three-attribute objects sharing two features in common with an unpresented three-attribute object (target) will produce an illusory perception of the target. We performed Experiment 2 to verify this prediction.

**Figure 2:**
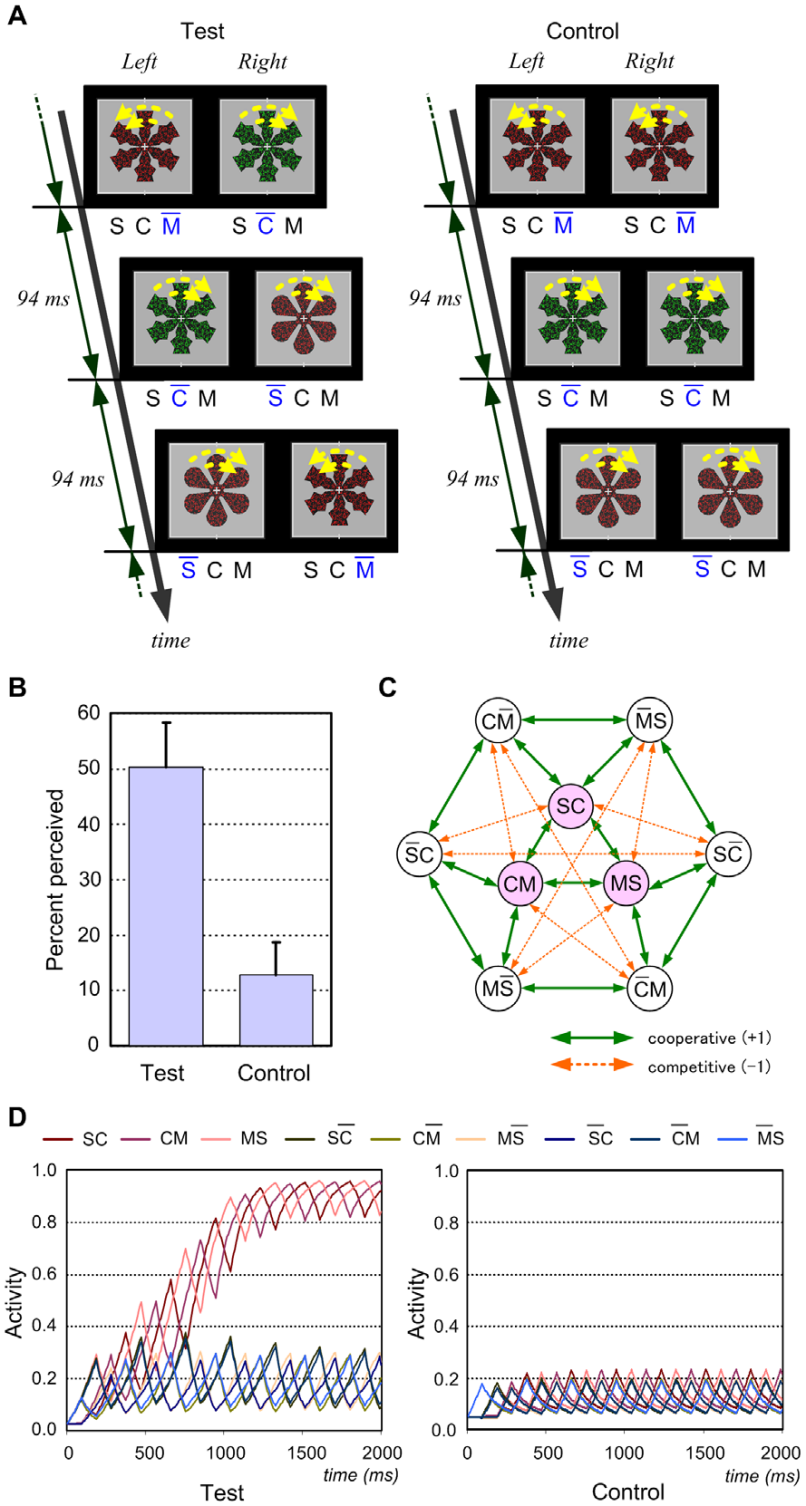
Illusory perception of multiattribute objects predicted by the paired-attribute model. (A) Example of stimuli used in Experiment 2. The target object denoted by SCM changed in each trial. S, C, or M with a bar denotes the distracting feature in the shape, color, or motion attribute, respectively. Subjects orally reported their view after a 3.3-s stimulus presentation. No feedback was provided for their answers. (B) Mean percentage of trials in which the reported shape, color, and motion were those of the target. Error bars indicate s.e.m. (*n* = 10). (C) Paired-attribute model accountable for the empirical result. Cooperative units are interconnected with connection weight 1, and competitive units with −1. (D) Simulated activities of individual units.

In each trial, a target was selected from among 8 three-attribute objects, and a series of stimuli was presented to an observer uninformed of the target (Figure 2A, see also supporting information Video S1). Each stimulus differed from the target in motion, color, or shape, and was presented for 94 ms.

In the control condition, the stimulus series was presented simultaneously to both eyes. Most observers perceived three objects in sequence, and the percentage of trials in which the target was reported was at the chance level (12.5%). In the test condition, the series was presented with a different phase to each eye and a stable view was perceived continuously without binocular rivalry. The target was reported in about half of the trials (Figure 2B); in most of the other trials, the target was recognized but one or two distracting features were also perceived. Although there were considerable differences between subjects, many subjects clearly perceived a three-attribute object in the test condition and did not notice that it was not actually presented to them.

These results do not support the all-attribute model, because competition between object representations cannot account for the findings that the unpresented target object was perceived and that no binocular rivalry occurred in the test condition in which different objects were always presented to both eyes. In addition, the illusory perception of the target is not accounted for by the predominance of target features over distracting features, because target features were also dominant in the control condition. It is also not a result of misbinding of individual features or illusory conjunctions [2], [18], which can be observed in typical rapid serial visual presentation tasks, because the target was barely perceived in the control condition.

A possible explanation for the observed difference between conditions might be that the illusory perception requires concurrent presentation of three target features occurring in the test condition only. This explanation, however, is inconsistent with the finding in Experiment 1 that an illusory three-attribute object was perceived in condition 2, in which three features of the object were never presented simultaneously. Thus, the experimental results are difficult to explain reasonably using any existing model or theory.

However, they do conform to the paired-attribute model. Let us assume for simplicity that two attributes are monocularly bound and that attribute-pair representations are binocular. Then the paired-attribute model can be demonstrated by the network shown in Figure 2C. Each unit of this network corresponds to a feature pair and receives an external activation signal when a stimulus containing the feature pair is presented to either eye. Different units have positive or excitatory interconnections if they correspond to different attribute pairs but share one feature in common (e.g., units SC and CM), and have negative or inhibitory interconnections if they correspond to the same attribute pairs and are mutually incompatible.

Mathematically, this network has an “energy” (or Lyapunov) function similar to the Hopfield neural network [19], which ensures that the network converges to a stable equilibrium state if the external signals are fixed. The number and distribution of stable states depend on the fixed external signals; when external signals are not sufficiently large or no units are sufficiently stimulated, only the state in which all units are inactive is stable. If three units that are mutually compatible receive a strong external signal, the state in which only these units are active is generally most stable. However, if all units equally receive a sufficiently large signal, the most stable state is that in which only the three units corresponding to a pair of target features are active, because these units have four positive connections from others whereas the other units have three (note that units corresponding to a pair of distracting features are excluded from this network because they are never activated in this experiment). A similar situation is considered to occur in the test condition in which all nine units are equally and frequently stimulated.

In fact, the model shows behavior as shown in Figure 2D. In the test condition, the three units SC, CM, and MS are activated to be predominant over the other units, implying that the target SCM has been recognized. On the other hand, they are not sufficiently activated in the control condition in which only three units are stimulated simultaneously and their activity decays until they are restimulated.

### Experiment 3

Another exclusive prediction of our hypothesis is that multiple attribute-pair representations are stored in memory, which could cause specific errors in a short-term memory task. We performed Experiment 3 to explore this possibility.

**Figure 3:**
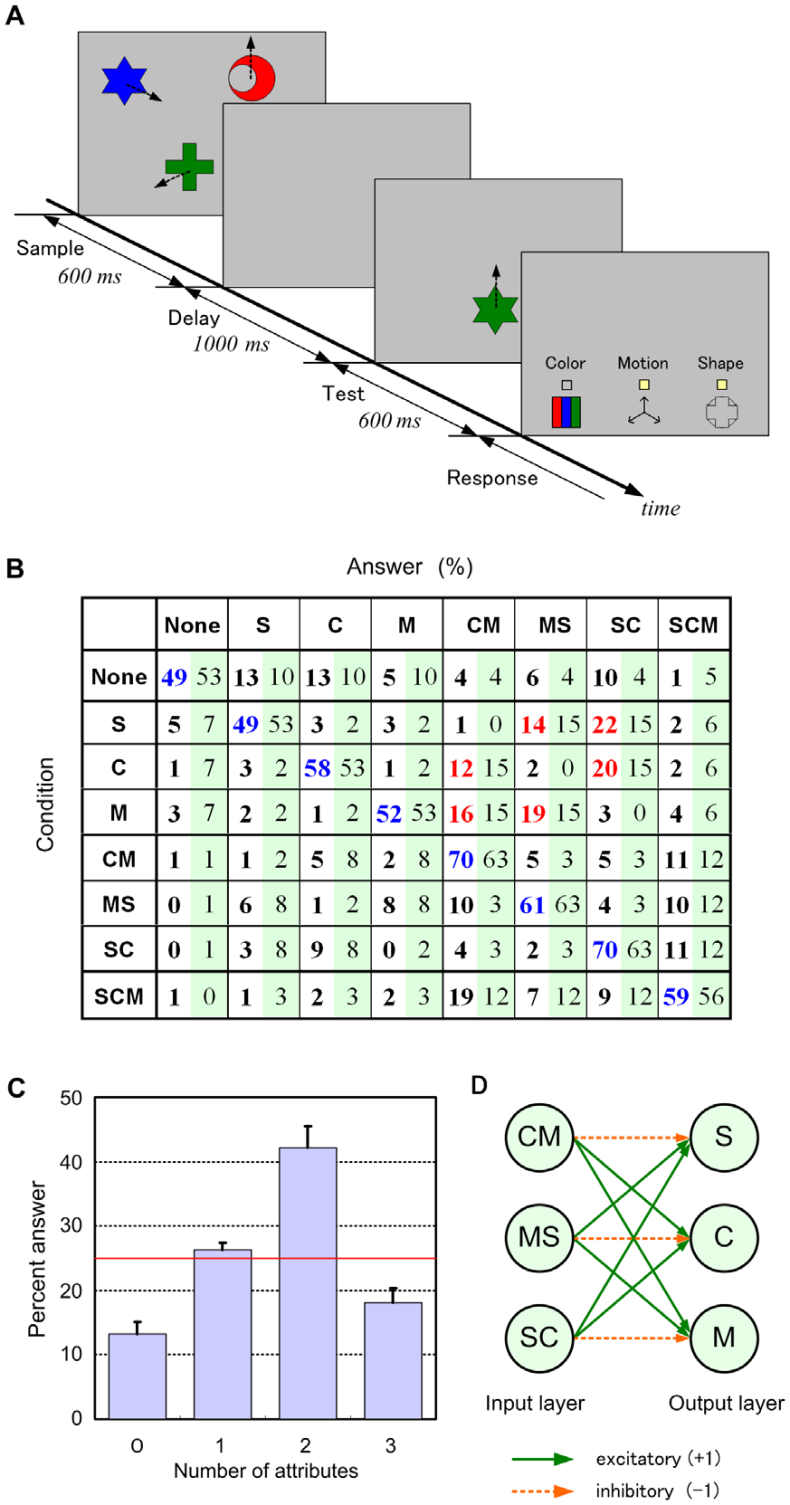
Empirical and simulation data from a change-detection task. (A) Procedure for a single trial in Experiment 3. (B) Distribution of subjects' answers for each case. The left value in each cell is an empirical percentage, and the right value is that calculated by the model in (D) with best fit parameters of *c* = 2 and *p* = 0.1. Blue numbers in the diagonal cells indicate correct answers, and red numbers indicate an illusory change in an attribute caused by a change in another attribute. (C) Percentage of the number of attributes judged to be changed. The red line indicates the actual percentage. Error bars indicate s.e.m. (*n* = 10). The differences between the two-attribute condition and the other conditions were significant (*P*<0.01). (D) Two-layer network for converting changes in individual attributes from changes in attribute pairs.

In this experiment, subjects viewed a sample display comprising 3 three-attribute objects, and after a brief delay, compared a test object with the sample object for shape, color, and direction of motion in the same location (Figure 3A, see also Video S2). Four conditions appeared randomly with equal probability: (0) no attribute changed (case None), (1) one attribute changed (cases S, C, and M), (2) two attributes changed (cases SC, CM, and MS), and (3) all attributes changed (case SCM).

Subjects' answers for each case were distributed as shown in Figure 3B. Subjects most frequently judged that two attributes had changed, although the actual frequency was equal (25%) in all four conditions (Figure 3C). Interestingly, when only one attribute changed, subjects frequently misjudged that another attribute had also changed (34% of trials, 74% of errors). In contrast, when two attributes changed, error trials reflecting the misjudgment that only one attribute had changed were not that frequent (11% of trials, 33% of errors). This finding indicates that a change in a single attribute often produces an illusory change in another attribute.

Again these results seem difficult to explain using the single-attribute or all-attribute models, but can be well accounted for by the paired-attribute model. The illusory change phenomenon can be understood based on change detection at the attribute-pair level.

More specifically, let us consider a simple two-layer network (Figure 3D) in which each unit in the first layer retains the corresponding attribute pair of the sample object and sends a mismatch signal to the second layer when the attribute pair of the test object differs from the retained memory. Each unit in the second layer detects the change in the corresponding attribute by computing the weighted sum of the mismatch signals. If retained memory is never lost, this network makes no or few errors equally in all conditions (the error rate depends on the scale parameter *c*). However, if the memory is lost with a certain probability *p*, the error distribution becomes biased and a distribution similar to the empirical data is obtained, as shown in Figure 3B.

## Discussion

The results of Experiments 1 and 2 indicate that parallel perceptions of multiple attribute pairs lead to recognition of a multiattribute object, and that object recognition is not necessarily based on unified object representations. They also suggest that binocular rivalry for multiattribute objects reflects competition mainly between incompatible feature pairs that are bound monocularly, which should impact the debate on whether binocular rivalry is based on eye or stimulus [10], [17], [20]–[22].

The results of Experiment 3 alone might not be sufficient evidence for our hypothesis that visual short-term memory stores paired attributes rather than integrated objects, but it is consistent with the results of a recent study suggesting that the unit of memory is a feature conjunction [14]. It can also partly account for the conflicting results of previous studies [11]–[13] because it predicts that memory capacity of objects will decline as three or more attributes are involved. Our model also indicates that a comparison between sample and test objects is performed in parallel for respective attribute pairs, which is consistent with the finding that a visual search for three-attribute objects or triple conjunctions can be faster than searches for two-attribute objects or standard conjunctions because the finding is considered to reflect parallel processes in a serial search [23].

We therefore conclude that our results support the paired-attribute model, suggesting that attributes of an object are integrated with one another to form multiple attribute-pair representations and that many cognitive processes are based on the network of these representations rather than unified object representations. We also consider that no more than two attributes are directly bound together to form a single representation, except for a limited number of very familiar objects, because currently there is no concrete evidence or indispensability for such total integration. For example, current evidence for “object-based” attention [8], [10], [24], [25] can be understood also in terms of “attribute pair-based” attention.

If our view is correct, the binding problem is greatly facilitated in computational theory, and many possible binding mechanisms can solve it. Then, the critical question is “what is the substance of attribute-pair representations in the brain,” rather than “what neural mechanisms are involved.”

Although the present study does not provide an answer to this question, we speculate that part of the neuronal population encoding an attribute is modulated by another attribute, and different parts are modulated by different attributes; thus, an attribute pair (e.g., shape and color) can be represented by two neuronal groups (“shape neurons” modulated by color and “color neurons” modulated by shape). An example of the population modulation presumed by us has been reported in a previous study [26], in which some neurons responding to a stimulus figure showed an abrupt decrease in activity when the color cue was switched. According to our computational theory, such a selective decrease in population activity (called “selective desensitization” in our theory) is a simple and reasonable method of integrating two types of information to evoke different actions depending on how they are combined [27].

To briefly explain the essence of this theory, let us consider a very simple model in which shape and color are encoded by different population of binary (±1) elements. Assume, for example, that shape 1 and shape 2 are represented by code patterns S_1_ = (+ + + + − − − −) and S_2_ = (+ − − + − + + −), respectively, and that color 1 and color 2 are represented by C_1_ = (+ + − − + + − −) and C_2_ = (− + − + − + − +), respectively. Then, an object with shape 1 and color 1 (denoted by S_1_C_1_) can be represented as the concatenated code pattern (S_1_, C_1_) = (+ + + + − − − − + + − − + + − −), and similarly for other objects. However, this concatenation is different from the binding we described in the Introduction, because the concatenated patterns cannot always be associated directly with arbitrary responses. For example, a generalized XOR problem, namely, associating objects S_1_C_1_ and S_2_C_2_ with response A, and S_1_C_2_ and S_2_C_1_ with response B, is unsolvable for an ordinary two-layer network. Although a three-layer network with a hidden layer can solve this problem, the required number of hidden elements increases in proportion to the number of possible combinations of shape and color.

However, we found that this problem can be solved without introducing hidden elements if each element in the first layer can be selectively desensitized to take a neutral value (0). Specifically, consider the case in which each “shape element” is desensitized if the corresponding “color element” is inactive (−). Then shape 1 modulated by color 1 is represented as a code pattern S_1_(C_1_) = (+ + 0 0 − − 0 0), shape 1 modulated by color 2 as S_1_(C_2_) = (0 + 0 + 0 − 0 −), and shape 2 modulated by color 1 or 2 as S_2_(C_1_) = (+ − 0 0 − + 0 0) or S_2_(C_2_) = (0 − 0 + 0 + 0 −), respectively. These patterns can be associated directly with arbitrary patterns if the number of elements is sufficient. In addition, they include enough information on both shape and color so that the original code patterns can be easily retrieved using a simple associative network. For this reason, the color signal is required only when the shape elements to be desensitized are selected, and connections between shape elements and color elements need not be direct or permanent. Thus, this theory provides a candidate mechanism of feature binding and a possible computational role of attention in it.

This speculation, however, requires further examination. Moreover, the paired-attribute model should be applied to many other cognitive processes and be tested to obtain direct evidence for our hypothesis. Nevertheless, we believe that our results will provide a key to the binding problem and other problems in cognitive science.

## Materials and Methods

### Ethics Statement

This research was performed in accordance with the Ethical Principles of Psychologists and Code of Conduct of the American Psychological Association. The experiments posed no danger of infringing human rights, and written informed consent was obtained from all participants. At the time this research began, institutional review board approval was not required.

### Experiment 1

The subjects were ten undergraduate and graduate students who had roughly the same eyesight for right and left eyes, did not have a squint or a very astigmatic eye, and were blinded to the experimental purpose. They all participated as paid volunteers and provided informed consent for participation.

Subjects viewed the stereo images through a stereoscope in a dark room. For each subject, mirrors of the stereoscope were carefully adjusted to ensure correct binocular alignment of the images with the central fixation cross and the square frame subtending 8.7 degrees, which were also presented during experimental trials to aid in the maintenance of proper convergence. The luminance of green was adjusted equal to that of gray (1.84 cd/m^2^), such that an alternate (10.6 Hz) green and gray presentation elicited a minimum perception of flicker. The luminance of red was adjusted equal to that of green in a similar manner. The background (outside the stimulus) was dark gray (0.45 cd/m^2^).

Subjects were instructed to hold down button A or B when stimulus A or B was perceived, respectively, and both buttons if both stimuli were partly perceived in different fields. They were also asked to avoid blinking intentionally during trials and to describe their perception after trials. For each condition, each stimulus was presented to both eyes with a notice of the type of stimulus (A or B) and the subjects orally reported their perception.

After practice trials, subjects performed 12 experimental trials of 60 s each: two trials in which stimuli A and B were presented to the left and right eyes, respectively, and two trials vice versa, for conditions 1, 2 and 3. These trials were divided into four blocks of three trials representing Conditions 1, 2, and 3; the order of conditions was fixed for one subject but counterbalanced across subjects.

The stimuli illustrated in Figure 1A are based on a circular pattern subtending 7.3 degrees composed of gray random dots (density, 0.23) on a black background. This pattern was also used as a mask presented before and after stimulus presentation. Displays S_A_ and S_B_ were generated by drawing black outlines and filling the inside with the dot pattern. C_A_ and C_B_ were generated by replacing gray dots in the pattern with green or red dots, respectively. M_A_ and M_B_ were generated by successively rotating the pattern in a clockwise or counterclockwise direction, respectively, by 0.4 degrees during each refresh period of 11.8 ms (34 deg/s). M_A_S_A_ and M_B_S_B_ were generated by rotating S_A_ and S_B_, respectively, and S_A_C_A_, S_B_C_B_, C_A_M_A_, C_B_M_B_, S_A_C_A_M_A_, and S_B_C_B_M_B_ were colored by replacing gray dots with green or red dots.

Each display in conditions 1 and 2 was presented repeatedly with an interval of 141 ms at 1.6 and 3.2 degree rotated positions, respectively, from the previously presented position, such that the dot pattern was not discontinuously rotated. Experimental parameters were determined by performing preliminary experiments with other subjects.

The obtained data were analyzed using repeated-measures ANOVA. The effect of conditions was significant for the data in Figure 1B and C (*F* [2], [18] = 12.9, *P*<0.001 and *F* [2], [18] = 15.6, *P*<0.001, respectively), and post hoc comparisons were performed using the Bonferroni test.

### Experiment 2

The same ten subjects from Experiment 1 participated in this experiment after Experiment 1.

Subjects were instructed to view the stimulus displays without blinks and orally report the shape, color, and direction of rotation that they perceived. Although subjects were asked to report as specifically as possible, unspecific answers such as “both flower and snow,” “either red or green,” and “unclear” were allowed. Each subject performed sufficient practice trials with no feedback and then performed 64 experimental trials in random order: 32 trials (four trials for each target object) for the test condition and 32 trials for the control condition.

Each stimulus illustrated in Figure 2A was generated in a manner similar to Experiment 1, but the combination of features was selected from among eight cases and changed in every trial in random order. Each trial started with a 0.5-s presentation of the mask, followed by a serial presentation of three stimuli repeated for eight cycles (2.25 s) with an interval of 282 ms at a 3.2 degree rotated position and ending with a 0.5-s presentation of the mask.

The data obtained were subjected to the paired *t*-test. The difference between the conditions was significant (*t* [9] = 5.02, *P*<0.001).

### Model Simulation for Experiment 2

Each unit of the model shown in Figure 2C receives signals from other units and an external activation signal, and emits the output *x_i_* according to the inner potential *u_i_*. The activation signal *s_i_* is 1 when a stimulus containing the corresponding feature pair is presented, and 0 otherwise. In mathematical terms, 
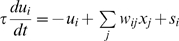
 and 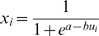, where *w_ij_* is the connection weight (1, −1, or 0), *ι* is the time constant of dynamics, and *a* and *b* are positive parameters. Parameters used in the simulation shown in Figure 2D are *a* = 3, *b* = 2, and *τ* = 100 ms.

### Experiment 3

A different set of ten undergraduate and graduate students with normal or corrected vision participated in this experiment. All were paid volunteers who were blinded to the experimental purpose and provided informed consent.

Subjects were instructed to watch a sample display, compare a test object with the sample object in the same location, and respond by clicking on icons using the mouse. To balance the tendency to hesitate from clicking on icons when the subjects were unsure about their judgment, we instructed them to mark icons corresponding to changed attributes in the first or last half (counterbalanced between subjects) of the trials and to mark icons corresponding to unchanged attributes in the other half.

Each subject performed sufficient practice trials with no feedback and then performed 600 experimental trials in random order (except for one subject, who performed 480 experimental trials): 150 (120) trials each for cases None and SCM and 50 (40) trials each for cases S, C, M, CM, MS, and SC.

The three objects in the sample display had different shapes (star, moon, and cross of the same size; 1.6 deg×1.6 deg), colors (red, green, and blue; 16.5 cd/m^2^), and motion directions (12, 4, and 8 o'clock at equal speed; 1.3 deg/s). The background was gray (11 cd/m^2^). These parameters were determined by conducting preliminary experiments with other subjects such that the percentage of correct answers would be almost independent of the type of attributes.

The obtained data were analyzed using repeated-measures ANOVA. The effect of attribute number in Figure 3C was significant (*F* [3], [27] = 24.2, *P*<0.001). Post hoc comparisons were performed using the Bonferroni test.

### Model Simulation for Experiment 3

Each unit in the first layer of the model (Figure 3D) emits 2 if both the corresponding attributes are changed, 1 if one of them is changed, or 0 if neither is changed. Each unit in the second layer emits 1, indicating that a change in the corresponding attribute is detected, with probability 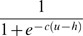, where *u* denotes the weighted sum of the input signals and *h* denotes a threshold or bias term (*h* = 1 in the normal case). We assume that the memory of an attribute pair is lost with probability *p*; when this occurs, the corresponding unit sends no signal and *h* is decreased by 1 (*h* = 1−*k*, when *k* memories are lost).

## Supporting Information

Video S1 Example of stimulus displays in the test condition of Experiment 2. The stimulus set is the same as that shown in Figure 2A. Although the frame rate, luminance, color balance, size, and other conditions may be considerably different from those actually used, the test trial can be experienced by fusing two stimulus images similar to the fusing done when viewing stereo images. Looking through a stereoscope (preferred) or two pipes for respective eyes is recommended. If you perceive a red snow shape rotating clockwise, you are seeing an illusion. You can also experience the control trial by viewing either image with both eyes. Note that because of individual differences and the difference in conditions, you might not see the illusion clearly.
(5.49 MB AVI)

Video S2 Example of stimulus displays in Experiment 3. Eight trials in conditions 1 and 2 appear in random order; after each trial, icons indicating changed attributes are shown, although in the actual experiment, trials in conditions 0 and 3 were also included, and the correct answer was not fed back to the subject.
(6.77 MB AVI)
